# Shedding light on those who provide support

**DOI:** 10.7554/eLife.64739

**Published:** 2020-11-23

**Authors:** Elsa Loissel

**Affiliations:** eLifeCambridgeUnited Kingdom

**Keywords:** mental health, academia, survey, supporters, None

## Abstract

An eLife survey explores the experiences of those in the research community who support colleagues struggling with their mental health.

As concerns about poor academic mental health grow (Levecque et al., 2017; Morrish, 2019), it is also becoming clear that certain individuals in the community try to help those going through a difficult time (Hughes et al., 2018; Technician Commitment et al., 2019). Yet these relationships remain poorly understood, putting them at risk of being overlooked by institutions.

In response, eLife collaborated with scientists to release, in late 2019, a survey that examined the experiences of those who support researchers struggling with their mental health. The final dataset captures the voices of over 1,500 respondents in various academic roles. The results – relayed in detail in a report (Loissel et al., 2020) – paint a complex picture of the needs and experiences of these ‘supporters’ (see appendix 1 for details of the dataset).

In this article, we share five main findings – as well as our dataset and code – for those who strive to improve mental health in academia to build upon. While many of the pressures that supporters experience are deeply embedded in the way academia currently operates, a better understanding of what these individuals do and need may help to pave the way for change. As the COVID-19 pandemic unfolds and exacerbates the academic mental health crisis (Chirikov et al., 2020), these discussions are urgently needed.

## Peers often support peers

**Figure 1.**
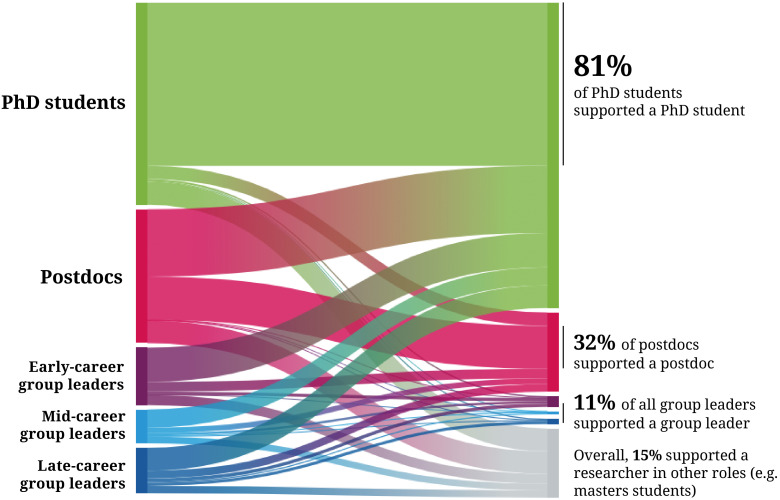
Alluvial diagram of the career stages of the individuals that PhD students, postdocs, early-, mid- and late-career group leaders support. The experiences of supporters (left) at five career stages were considered: PhD students (green), postdocs (red), early-career group leaders (less than five year since independence; purple), mid-career group leaders (between five and ten years since independence; light blue) and late-career group leaders (more than ten years since independence; dark blue). The career stage of the person they last supported (including individuals at other career stages; grey) is reported on the right. The majority of PhD students and a large proportion of postdocs support someone who is at the same career stage. However, those with official leadership roles mostly provide help to PhD students and, to a lesser extent, to postdocs. Only a minority of group leaders provided support to another group leader, despite 47.4% of early-career group leaders, 37.8% of mid-career group leaders and 23.9% of late-career group leaders reporting they were themselves struggling with their mental health at the time of support.

Our results indicate that, at least for our sample, supporting colleagues who struggle with their mental health is a common occurrence in academic settings: about two thirds of respondents have already helped more than two individuals. These interactions mainly take place between peers at the same career stage – especially for PhD students and postdocs – or flow from an established to a more junior researcher (Figure 1). Many researchers, including group leaders, also provide support to colleagues who they have no official responsibility towards. "It's a thankless job but somebody has to do it," said one postdoc. "Providing secondary mentorship (disciplinary, hidden curriculum stuff, and emotional) is critical to making the ‘trains run on time’."

Failure to acknowledge that help is offered by many populations, including peers and non-academic staff, runs the risk of leaving a large proportion of these informal supporters without the backing they require. "I had no outside assistance," said one respondent. "I don't know if there are particular guidelines in place for assisting individuals who assist others with mental health problems. As a postdoc (a long-term one), I am excluded from these types of information workshops because I am not a 'supervisor'."

Our results also highlight that only a minority of supporters help early-career group leaders, despite this population also reporting being under considerable pressure (Lashuel, 2020). "I wake up every day feeling overwhelmed and I often have upsetting situations and almost no one to talk to about it," replied one early-career group leader. "This is the worst time of my academic career and of my life right now."

## Complex needs, complex support

The survey responses paint a picture of in-depth, long-term relationships that involve several types of support and address a range of mental health issues – at least as perceived by supporters. About two thirds felt that the individuals they helped were struggling with depression/low mood and anxiety, but some also reported supporting individuals who experience conditions associated with high-risk for harm, such as sleeping problems (29.5%), suicidal thoughts (16.1%), self-harm (6.5%), substance abuse (7.3%) and eating disorders (6.1%). Supporters also believed they had helped individuals whose conditions were, as far as they knew, connected to traumatic events that had taken place in academic settings, such as bullying (20.9%), sexual assault or harassment (5.4%), and racism (7.7%). Despite the potential severity of these conditions and experiences, almost half of supporters had helped someone who, at least at some point in the relationship, was not receiving professional help.

Nearly all supporters provided emotional support, but over half also suspected someone needed help, and proactively encouraged this person to open up. Many also gave advice about how to manage one's mental health (46.6%) and about resources that could be accessed (50.6%). Supporters also provided practical help – often with work (41.7%) – and almost a quarter advocated for a colleague, such as requesting additional help on the person’s behalf. These supporting relationships were also not short-lived: most lasted for over six months, and about a fifth for over a year.

## A positive experience, but poorly supported

The majority of respondents found that helping someone was a positive experience – "one of [my] most rewarding experiences in grad school", as one PhD student put it – and most felt appreciated by the person they had helped. For one respondent, the "pursuit of knowledge is meaningless if uncoupled from a sense of shared humanity with our colleagues."

**Figure 2.**
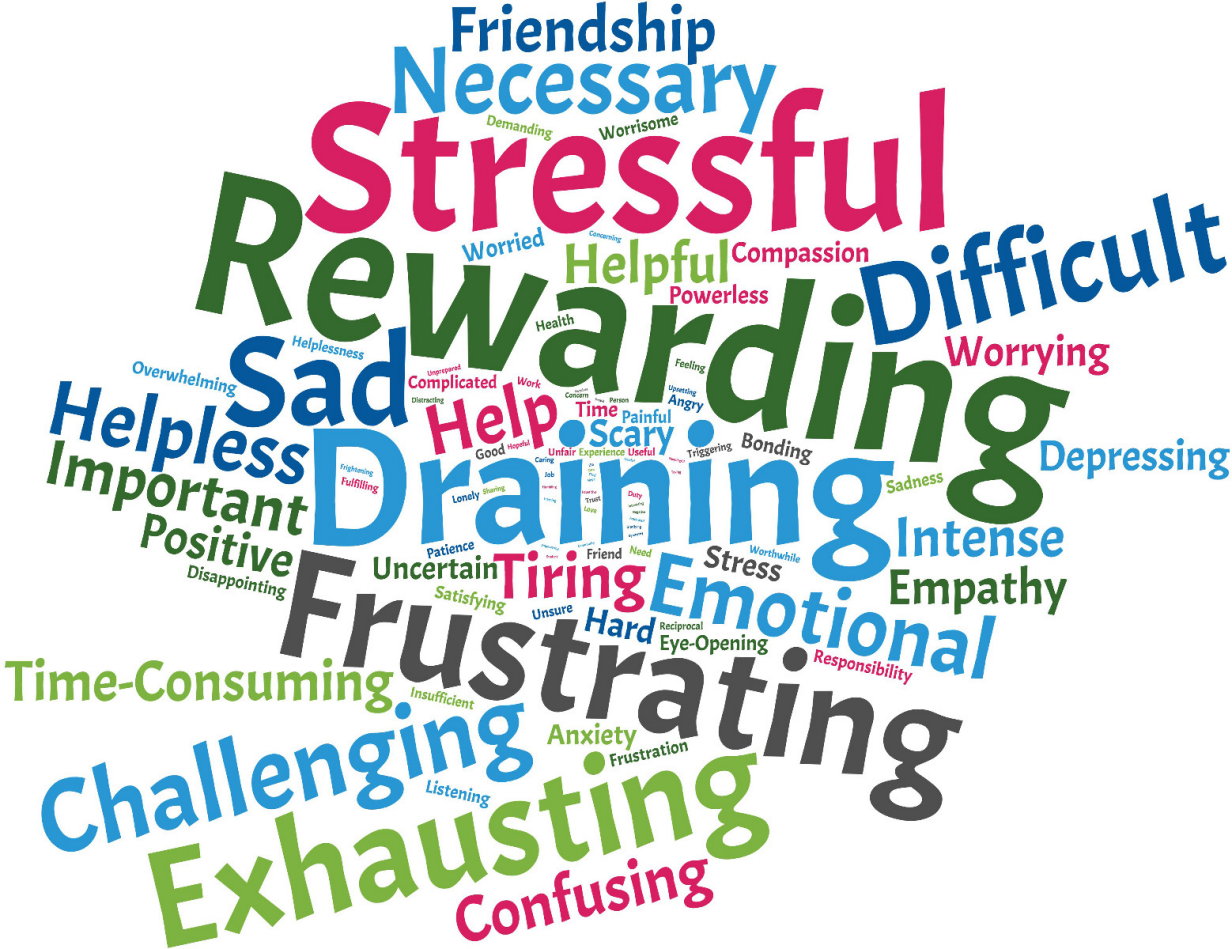
How supporters feel about helping someone else. Word cloud generated based on answers to the question "Please list up to five words to describe your [supporting] experience".

Yet, 76.1% of supporters felt this role was emotionally draining or stressful (Figure 2). "We were not prepared to deal with the issues", recalled one postdoc, "and we were constantly scared of giving the wrong advice, and the repercussions that [it] could have." It is not surprising, then, that only about a quarter of supporters reported not needing emotional support – with nearly 20% needing but not accessing this type of help. Family, partners and friends outside of academia were the main source of emotional support, suggesting that work issues spill over to the personal sphere and that institutions may be failing to fulfil that need. "My department sent one email, last year, which essentially told lecturers off for not 'practising self-care', and gave the reason why we should do so as [being] that students need us to be good role models", recalled one PhD supervisor. "That email actually made me cry with frustration. I feel angry, inadequate and exhausted all the time. I'm just expected to fix the problems with no thanks or support, no one to turn to, and the threat of complaints if I don't manage it."

Only about half of supporters were confident they would say or do the right thing, and not knowing what to do was the main reason for not having supported someone in need. Only 11.5% had already received practical information to guide their supporting roles. While over half needed this type of advice, just 9.8% accessed these resources without any issues — lack of time being the main barrier. What supporters wanted most was advice from professionals and mental health training such as workshops or courses, with some commenting that their own therapists had been a precious source of information. And indeed, supporters mostly turned to other people for practical advice, with friends and family being the first port of call, before colleagues.

## Certain populations of supporters face specific challenges

Across the board, women who were supporters faced additional challenges compared to men. At the time of support they were more likely to be helping several people, to be struggling with their own mental health, and to be feeling less supported or valued by their institutions for helping someone. Women were also more likely to report that other people approached them because they were known as supporters. "I am repeatedly frustrated by my (often male) colleagues' stated belief that 'there is no mental health problem' at our institution," said one female mid-career group leader. "They don't know about it because their trainees come to me, not to them, with their issues. I receive no institutional recognition for this role."

In parallel, women felt their supporting role was more emotionally draining or stressful, more time-consuming, and impacting their personal lives and work more compared to men. Further analyses are needed to confirm these results and disentangle how these experiences emerge, interact and reinforce each other: yet these findings are consistent with literature on how gender influence academic experiences (El-Alayli et al., 2018; Guarino and Borden, 2017; Sprague and Massoni, 2005).

“I am repeatedly frustrated by my (often male) colleagues' stated belief that 'there is no mental health problem' at our institution. They don't know about it because their trainees come to me, not to them, with their issues. I receive no institutional recognition for this role.”

**Figure 3.**
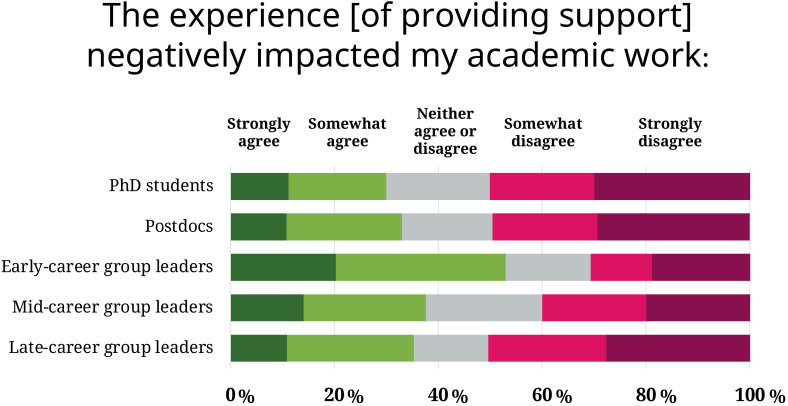
Impact of the supporting experience on the supporters’ work, by career stages. Compared to other career stages, group leaders with less than five years of experience are significantly more likely to report their work being impacted by their supporting relationship. Only supporters who were PhD students, postdocs or group leaders at the time of support are considered for this graph.

Overall, those new to leadership positions also emerged as a group with specific needs; compared to individuals at other career stages, they were more likely to find that supporting someone had an impact on their work and left them drained or stressed (Figure 3). Navigating a supporting relationship as first-time supervisors could come with specific challenges, such as learning how to professionally manage a distressed junior colleague, and the resulting group dynamics. "I was afraid I was providing the wrong kind of support, was afraid I would be blamed as the cause for additional stress", recalls a mid-career group leader. "What was expected out of [the distressed junior colleague] was low, but the illness was not known to the group. It demotivated the group as others thought I did not have uniform standards for everyone."

**Figure 4.**
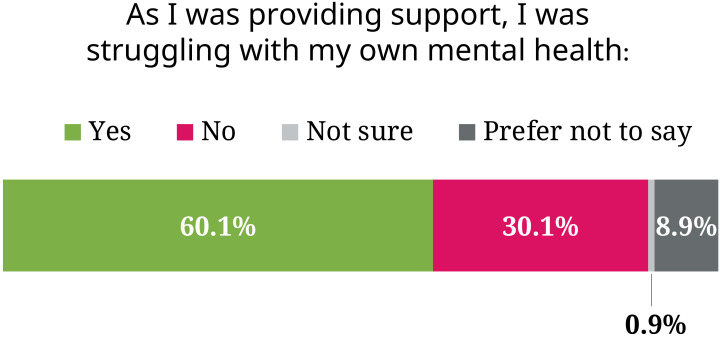
Many of those providing support were struggling with their own mental health. 60.1% of respondents answered "Yes" to the statement: "As I was providing support, I was struggling with my own mental health".

Over three quarters of respondents reported having gone through times when their mental health was poor, suggesting that our sample is enriched in people having faced mental health issues. "It is generally the ones who struggled with mental health themselves who step up and care", remarked one postdoc, "but they need help, too." This also means that many were struggling themselves while providing support (Figure 4) – 83.8% of PhD students were in that position, for instance, and 23.9% of senior group leaders. This overlap between those who need and provide support may suggest that information on how to appropriately help others should be included in initiatives designed for researchers who experience mental health issues.

It would be crucial to also explore whether supporters with first-hand experience of poor mental health or who belong to underrepresented communities are more likely to provide support, and experience it differently compared to other groups. "This feels especially hard as a woman of color with my own mental health struggles who tries to be supportive to other women of color", said one PhD student. "I ended up playing supportive roles because people could not access mental health services." Understanding these interactions is particularly important since mental health issues may disproportionally affect populations already marginalized in higher education (Jochman et al., 2019; Semlyen et al., 2016; Ståhlberg et al., 2016; Stebleton et al., 2014). This could potentially triple the invisible workload of people who live with these health conditions, are underrepresented, and support other at-risk individuals.

## Structural barriers hinder support

**Figure 5.**
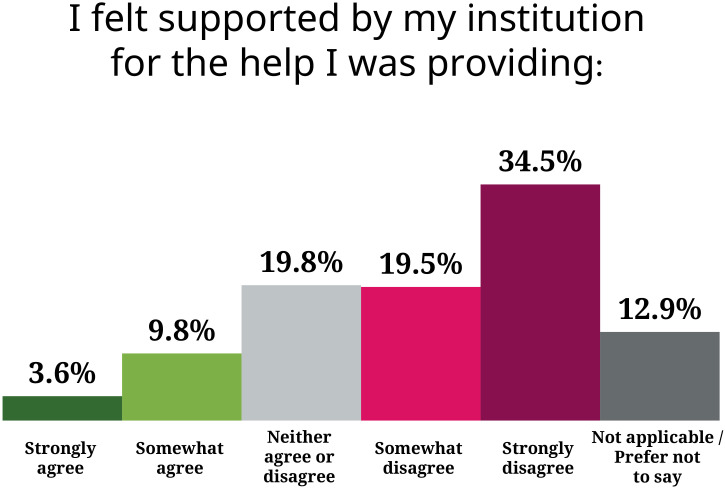
Support from institutions. The majority of supporters somewhat or strongly disagreed with having felt supported/valued by their institution (e.g.managers, department) for the help they were providing.

While about a third of those providing support said it had affected their work, just over half reported that it was taking a lot of time. Yet only 3.6% strongly agreed that their institutions valued or supported what they were doing (Figure 5). "Universities need to acknowledge this as work, for example in workload models, professional development or promotions discussions" said one university lecturer. "'They' love and need us to do it but don't support it. Many mental health supporters around me are fed up and burning out." Not valuing the help provided by supporters may lead them to worry their efforts are viewed negatively – one mid-career group leader recalls hearing: "Why are you doing all this work? They just need to go see a therapist!"

Most have also faced situations where they did not support someone who needed it. In particular, 49% of respondents cite struggling with their own mental health as a reason for not providing help: "It becomes difficult to be a good mentor for others when you are dealing with your own mental health problems" commented a group leader. Yet, 39.2% also explained finding that the person was difficult to approach. "Stigma is everything", explained one postdoc: "It is very difficult to offer support openly since most people prefer to hide. I understand very well this feeling." A PhD student concurred: "I wished that there was less stigma associated with needing and getting help. It's hard to seek or accept help, or tell others to seek and accept help, when it is still seen as a weakness." In turn, the weight of stigma could amplify confidentiality issues, making it more difficult for supporters to manage the supporting relationship and to look for help for themselves.

The hierarchical nature of the research community may have also stifled support: just over a fifth of supporters did not provide help for fear it would put them in a difficult position. One postdoc put it bluntly: "They were being bullied and I thought I would get bullied too." In fact, a number of respondents regretted not standing up for a colleague, especially in cases of abuse. "I wish I could have been an intermediary between them and the PI", recalled a respondent who was a postdoc at the time, **"**but that puts the 'helper' in a potentially bad situation."

Finally, many respondents noted in their comments that the culture of modern research, such as a relentless need to publish, large numbers of fixed-term contracts, inflexible funding deadlines and heavy workloads ultimately fosters an unhealthy environment that hinders support as well as creates mental health issues. One early-career group leader summed it up plainly: "Given that ideas, papers and grants are the metrics for success, is it any wonder that trainee mental health is sacrificed?". The COVID-19 pandemic, which has delayed many research projects, increased demands on teaching staff, led to hiring freezes, and placed additional pressures on those with caring responsibilities, may worsen this situation.

## What next?

Ensuring that all supporters – not just group leaders – receive help, training and recognition may be key to fostering healthy, positive relationships between those who provide and receive support. At present, the practical and emotional help that supporters need is found outside the institution, or it falls on colleagues – potentially raising confidentiality issues for the supported individual. Our results may highlight the need to go beyond one-off training sessions and provide on-going help for supporters, especially since most turn to conversations with other people for advice, rather than other types of resources. Such help and support also needs to take into account the full range of conditions and problems found in the research community: "I think people are becoming better at discussing overall depression and anxiety", said one PhD student, "but they still struggle to discuss suicide, self-harm, hallucinations, and other areas of mental health they perceive as 'more serious'."

However, unless the root causes of poor academic mental health are addressed, these measures could, in the words of a respondent, be "sticking plasters" at best. Ultimately, no amount of training or awareness-raising can change the fact people may not have space, time, and emotional resources to actually provide support. And while a collegial approach has some advantages, relying on researchers to monitor and be responsible for the mental health of their colleagues also raises issues. As a PhD student remarked: "It's important that this does not become a thing where people who are struggling need to out themselves in order to receive help. They are entitled to maintain their privacy and not to have to discuss this with their colleagues unless they want to."

Our results raise many questions, and we hope the community will criticise and build upon this work (see appendix 1 for resources). In particular, the factors which may predispose certain individuals to become supporters (such as gender or minority status), or which could make this invisible workload more difficult (such as supporting someone who is not helped professionally) should be carefully assessed. Supporting relationships will also need to be re-examined through the lens of the 2020 pandemic: in a socially-distanced, remote working environment, how will informal peer-to-peer support take place? Who will spot early, subtle signs of distress? Who will have the emotional bandwidth to answer them?

In the short-term, we hope our results will bring the work of supporters to the attention of institutions, and encourage reflection on how to value and support these individuals. A first step could be to better understand supporting relationships at the local level (e.g. in a department) to assess specific needs, create tailored solutions and evaluate the impact of interventions. 

We also wish for the survey to empower supporters, helping them break the isolation they may experience, and encouraging peers to enquire about the wellbeing of individuals who provide help as well as of those who need it (Box 1). And most importantly, for those who struggle, we hope they hear the voices of supporters who, in their open answers, reaffirmed their commitment to help, their sadness at seeing peers struggle, and their deep-set belief that, in the words of one respondent: "The presence of a mental illness does not impair an individual's ability to do great science."

Box 1. In their own words.

The following selection of responses illustrates some of the main messages to emerge from the survey.

"I wish there was someone to talk to who I can trust and will help me be a better and more supportive mentor."

"I definitely see a gender bias towards women taking time to support people when it is not part of their formal role. That is particularly acute when the group leader is not able or willing to offer the support needed: it often falls to senior female postdocs or research managers."

"Due to the stigma of mental illness the situation was not disclosed to other members of the lab so I felt alone on my role."

"How do you add yet another item to the list of things that advisors must do? We are not trained nor selected to do this work. What about those of us uncomfortable or unable to make these observations or intuit what is going on internally for our trainees? I think it's crazy to expect us to handle these situations like professionals. On the other hand, we are in the best position to do just that! I wish there were a way that graduate programs and departments took on this role with people who are truly qualified to manage other's mental health instead of adding to the huge list of responsibilities for PIs."

"It takes a moment of kindness for people to feel comfortable talking to you. A smile, a greeting or acknowledgement of their existence speaks volumes."

"Only diamonds are made under pressure; good scientists are made with love and support."

## Note

This Feature Article is part of a collection on Mental Health in Academia.

Read more about supporters’ experiences as lab managers, postdoctoral students, technicians, and their role in academia.

In the UK and Ireland, the Samaritans can be contacted 24/7 on 116 123 or by email at jo@samaritans.org; in the US, the National Suicide Prevention Lifeline is 1-800-273-8255 and in Australia the crisis service Lifeline is 13 11 14. Please visit www.befrienders.org to find other international helplines.

## Appendix 1

Details of the survey sample The final dataset includes 1889 respondents, the vast majority of which reported having helped at least one person: over two-thirds worked in the life sciences and biomedical fields, with the humanities (9%) being the second largest group. 62% of respondents identified as women, 36% as men, and 1.3% as non-binary, gender-fluid or two-spirit. About a third identified as belonging to a group that was underrepresented in academia because of ethnicity, sexual orientation, disability status, or socio-economic background. At the time they provided support, 36.0% were PhD students, 23.8% were postdocs and 24.4% were group leaders; other respondents include undergraduate and Masters students, as well as other academic or non-academic staff. The majority of supporting interactions took place in the United States, the United Kingdom or the European Union. We alert readers to the fact that, as with most voluntary surveys, this work probably suffers from self-selection and sampling biases. We acknowledge that we did not capture the voices of all supporters: only those who could be reached, who recognized their interactions as providing support, and potentially who saw these experiences as worth reporting, for positive or negative reasons. Link to resources The survey received ethical approval from the University of Cambridge (2018-19/35). The full quantitative analysis of the survey is available in a report (Loissel et al., 2020), which also details the recruitment process. In addition, the following materials have been made available under a CCBY 4.0 license for others to reuse; the anonymised dataset; the R code used in the analysis; the survey questionnaire. Individuals wishing to find resources for their supporting roles can refer to the list we have curated in our report.
